# Integrated Design and Scheduling of Hydrogen Processes under Uncertainty: A Quantile Neural Network Approach

**DOI:** 10.1021/acs.iecr.5c03288

**Published:** 2025-10-22

**Authors:** Lavinia M. P. Ghilardi, Gabriel D. Patrón, Antonio Alcántara, Calvin Tsay

**Affiliations:** † Department of Computing, 4615 Imperial College London, London SW7 2AZ, U.K.; ‡ Sargent Centre for Process Systems Engineering, Imperial College London, London SW7 2AZ, U.K.; § Department of Statistics, 16726 University Carlos III of Madrid, Leganés 28911, Spain

## Abstract

The design and scheduling of electrolysis-based hydrogen production plants are subject to uncertainty in future electricity price predictions. Two-stage stochastic programming, which can model this uncertainty, often suffers from high computational costs. In this work, we propose the use of quantile neural networks as surrogates to model the so-called “second stage” for the integrated design and scheduling problem of a hydrogen process. Our surrogate model captures the distribution of the second-stage value function conditioned on electricity price-dependendent first-stage decisions. The neural network surrogate is then embedded into the two-stage stochastic program, enabling solutions to be found without computationally expensive sampling approaches. As the surrogate model outputs a distribution of the second-stage value function, we show this approach further enables joint optimization based on expectation and conditional value at risk, which is a measure of tail risk. Our results show that the inclusion of risk measures leads to higher investments in the electrolyzer and the storage to hedge against high electricity cost scenarios. The surrogate-based approach yields high-quality decisions and requires significantly less computational resources than the conventional sample average approximation.

## Graphical abstract

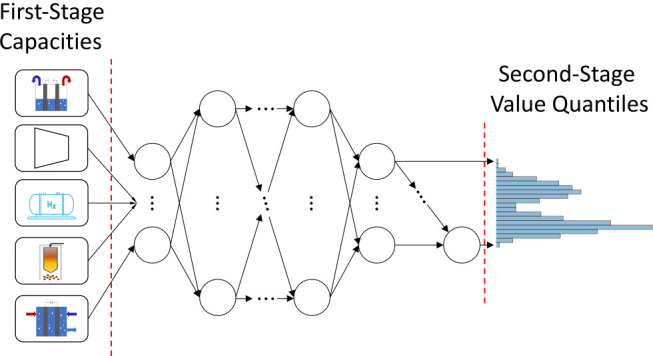

## 1 Introduction

Global renewable energy capacity is projected to nearly triple by 2030.^1^ While renewable generation sources have the potential to help decarbonize the economy, their intermittent nature, characterized by fluctuating supply, remains a critical challenge toward increased adoption.^2^ This supply intermittency often requires support from polluting baseload power sources or so-called ″peaker″ plants to maintain grid stability. With the expected rapid growth in renewable generation, developing efficient and scalable energy storage solutions is essential to reliably harness these sustainable resources and thereby reduce reliance on traditional carbon-intensive energy production.^2^

Among the various options for energy storage, hydrogen has received significant attention owing to its high mass-energy density, simple reaction chemistry, and flexibility for use as a feedstock and fuel.^3,4^ Among several production routes,^5^ hydrogen can be made in an electrolytic cell where water is split into component oxygen and hydrogen gases using an electrical current; the resulting hydrogen is called “green” when the electricity comes from renewable sources.^4^ This green hydrogen can then be used for short- to medium-term energy storage and converted back to electricity in a fuel cell or turbine.^6^ Alternatively, hydrogen can be compressed or kept in reservoirs for long-term storage, used as transport fuel, used for ammonia production, or deployed as feedstocks in heavy industry.^7^ The variety of options for downstream use is attractive to policy-makers, and green hydrogen has been included in many national energy development strategies^8^ including in the UK^9^ and the US.^10^ Despite its potential, installed hydrogen capacity remains low compared to future projections;^8^ hence, optimal design and operation strategies (in addition to technologial advances) play a key role in finding cost efficiencies that enable deployment at scale.

From a design perspective, the capacity of a hydrogen asset (i.e., electrolyzer, fuel cell, storage) must be sized given anticipated demand and energy prices. This must balance the trade-off between ensuring sufficient capacity to satisfy demand (whether for hydrogen or energy storage), and minimizing the capital cost.^11–13^ From an operational standpoint, hydrogen production systems can be operated following demand response when connected to power grids, i.e., dynamically adjusting energy consumption or production according to the price of electricity and grid demand. This paradigm is an apparent “win-win,” with operational flexibility improving the emissions/cost of hydrogen production^14–16^ while also providing much-needed energy storage to the power grid. In the context of hydrogen, demand response scheduling has been proposed to manage fuel stations^17,18^ hybrid storage systems^19,20^ and microgrids.^21–23^ Despite the success of demand response scheduling at large, it relies on accurate forecasts of future energy prices, which contain significant uncertainty in practice. Moreover, the design and operational decisions must be solved in an integrated manner, to produce optimal designs for *flexible* hydrogen production.^24,25^

Stochastic optimization enables distributions over uncertain parameters to be incorporated into mathematical programming formulations.^26^ Following the two-stage stochastic programming formulation, decisions are partitioned into first-stage (here-and-now) and second-stage (wait-and-see) decisions: the former are implemented at the time of optimization, while the latter can be implemented at a later time after the values of uncertain parameters are revealed. In other words, while the here-and-now decisions are deterministic, the wait-and-see decisions are conditioned on uncertain parameter distributions and allow for recourse once these uncertain parameters are realized in practice (i.e., drawn from their distributions). Stochastic demand response scheduling has been used as a tool to abate the economic suboptimalities in hydrogen storage systems induced by uncertainties in wind^27–31^ and photovoltaic^29–31^ power generation, as well as more directly through electricity demand^29–31^ and price.^29,30^ Generally, stochastic problems are formulated and solved based on expected values over uncertain distributions. Nevertheless, operators may also be interested in incorporating the notion of risk explicitly into optimization formulations. To this end, Rockafellar and Uryasev^32^ introduced a multiobjective formulation to jointly optimize expected values and risk, modeled using conditional value-at-risk, or CVaR. Risk-averse formulations have been deployed in hydrogen-electric microgrids with^17,33^ and without^34^ hydrogen vehicle fuel stations.

Despite the success and widespread popularity of stochastic optimization, it remains a computationally expensive problem to solve. For a stochastic program to be formulated and solved in closed form, the uncertain parameter distributions are often discretized into realizations (scenarios) of the uncertain parameters using the sample-average approximation, or SAA. The interested reader is referred to Kim et al.^35^ for a comprehensive review of SAA approaches. The fidelity of this approximation to the original continuous distribution is dependent on the number of samples, or scenarios; however, computational effort also scales with the number of samples, as more copies of the optimization model must be included, producing a large-scale *monolithic* problem. This limits the applicability of stochastic optimization to settings where the model is small, extensive computational resources can be used (i.e., offline uses), and/or few optimization runs are needed. Various algorithmic improvements have been proposed,^36^ such as decomposition approaches,^37^ dynamic optimization-based reformulation,^38^ and scenario reduction strategies.^39^

More recently, machine learning (ML) methods have been employed to accelerate stochastic programming. These studies largely focus on identifying a smaller set of representative scenarios or use ML surrogates to approximate the second-stage problem. In the former direction, Bengio et al.^40^ predict representative scenarios that allow for near-optimal primal solutions. However, their method relies on problem-specific heuristics for generating representative scenarios during training. In the latter direction, neural network surrogates have recently been proposed to model the second-stage objective in stochastic programming.^41–43^ The surrogate problem therefore optimizes the first-stage tactical decisions, given the first-stage cost and the neural network approximation of the second-stage objective. These approaches remove the trade-off between model size and number of SAA scenarios, thus resulting in a parsimonious formulation. In this area, Patel et al.^41^ first propose to embed trained neural networks via mixed-integer programming formulations to approximate the expected second-stage objective. This approach is further enhanced^42^ by improving the training via an adaptive sampling technique. These second-stage surrogate approaches focus on a central tendency approximation, preventing their application to risk-averse settings concerned with the tail of the distributions. To overcome this, Alcántara et al.^43^ propose the use of quantile neural networks (QNNs) as surrogates for the second-stage objective. This enables the learning of the entire wait-and-see distribution as shown in Figure 1, e.g., both expectation and risk are modeled by the surrogate. An additional advantage of this method is that it does not require scenarios as direct inputs to the ML surrogate. As a result, this approach does not suffer from scenario-related scalability in the data generation or in the formulation as in previous approaches.^41^

**Figure 1:**
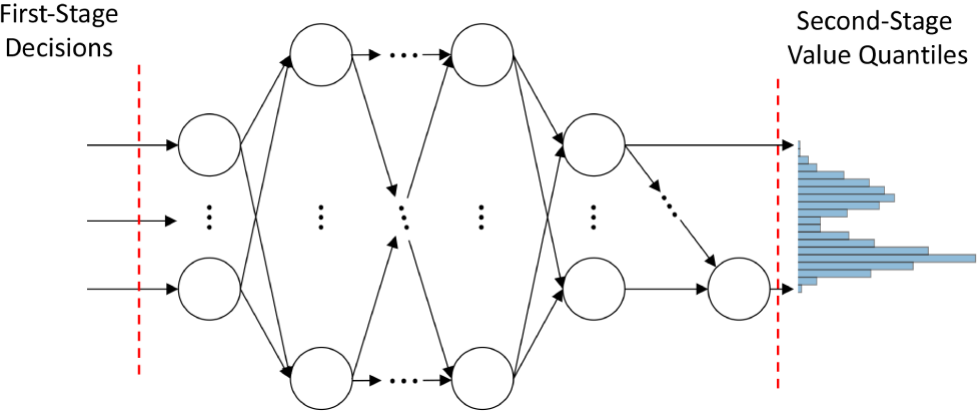
Overview of second-stage surrogate.

In this work, we present a QNN-based stochastic programming framework for integrated design and scheduling of energy storage processes, considering tail risk. Specifically, we present a joint risk-averse design and scheduling framework that enables optimal hedging against uncertainty in electricity prices and efficient energy systems scheduling. We consider the risk-averse optimization for an integrated hydrogen system (IHS) as a prototypical example. This falls into an important area of using ML approaches to aid in the optimization of IHS processes.^44,45^ The IHS can purchase and sell electricity from and to the grid and is connected to a downstream process with a fixed demand that must be continually satisfied. In this setting, the dynamic trajectory of the energy prices is uncertain, as predictions may be inaccurate at optimization (i.e., capital investment) time. The two-stage stochastic optimization problem for the IHS is solved with design (i.e., unit capacity) first-stage decisions and operational (i.e., electrical charge and discharge) second-stage decisions.

The remainder of this work is structured as follows: Section 2 presents our methodology including two-stage stochastic optimization, CVaR modeling, QNNs, and trust-region filters; Section 3 provides the IHS case study model, Section 4 presents results and analysis of the surrogate modeling methodology on the IHS case study; Section 5 provides conclusions and future directions.

## 2 Methodology

### 2.1 Risk-Averse Two-Stage Stochastic Optimization

Two-stage stochastic programs (TSSPs) aim to find a first-stage solution that is optimal for a distribution of possible realizations of the random uncertain variable(s) in the second stage. In the first stage, “here-and-now” decisions *x* are initially made on a tactical level, i.e., planned given the inherent uncertainty. Then, the realization of some uncertain parameter(s) ξ is revealed, affecting the course of the initial planning. In the second stage, “wait-and-see” operational decisions *y* represent the recourse actions taken to adapt to the particular uncertainty realization. Given the above, the decision variables of the original optimization problem are partitioned into vectors *x* and *y*. For the case of energy storage systems, we consider the future price trajectory of electricity as the main uncertainty ξ. The first-stage variables *x* must be decided before these prices are revealed and include equipment sizing, block electricity contracts, etc., and their associated costs. The second-stage decisions *y* correspond to the adjustment of operating schedules once electricity prices are known, or better anticipated.

Without loss of generality, we consider here the minimization sense, i.e., to minimize costs (or negated profit). In the risk-neutral setting, the goal is to minimize the total expected cost, which includes the deterministic first-stage objective *c* ^*T*^ *x* and the expected value of the second-stage cost, including recourse action.^26^ The classical formulation of a two-stage stochastic program is

1 $$\underset{x\in X}{\min}E\left[G\left(x,\xi \right)\right]=\underset{x\in X}{\min}c^{T}x+E\left[V\left(x,\xi \right)\right]$$

where *G*(*x*, ξ) is the overall value function, which is decomposed into a first-stage cost and the second-stage value function, denoted as*V*(*x*, ξ). The first-stage cost is deterministic, e.g., cost of capital expenditure, and assumed to be linear. The second-stage value function is further expressed as the solution to the second-stage recourse problem:

2 $$V\left(x,\xi \right)=\underset{y\in Y\left(x,\xi \right)}{\min}v\left(y,x,\xi \right)$$

In this formulation (eqs 1 and 2), $x\in R^{n_{x}}$ denotes the first-stage decisions constrained to the feasible set X and with associated costs $c\in R^{n_{x}}$ . The vector ξ ∈ Ψ denotes the random uncertain parameters assumed to follow some probability distribution P with support Ψ. Finally, the second-stage decisions are denoted as $y\in R^{n_{y}}$ and constrained to the conditional (i.e., decision-dependent) feasible region $Y\left(x,\xi \right):X\times \Psi \to R^{n_{y}}$ , which is taken to be a closed polyhedral set defined by the process model. Accordingly, V(x,ξ):X×Ψ→R maps the first-stage decisions and the uncertain parameter values to the second-stage value function. In this work, we assume that for every feasible x∈X and for every possible realization of ξ, the set Y(x,ξ) is nonempty. This assumption is commonly adopted in surrogate-based modeling for stochastic optimization^41,42^ and in the stochastic optimization literature more broadly.^46,47^ It can also be addressed by penalizing the deviation from feasibility in the recourse problem.^47^ In energy storage systems, the nonempty conditional feasible region assumption holds in many settings, as a feasible operating plan (e.g., constant or zero production) trivially exists for any installed plant capacity.

As the risk-neutral formulation focuses solely on the expected cost, it may fail to account for rare, but costly outcomes. To address this, risk-averse formulations incorporate measures such as Conditional Value-at-Risk (CVaR) to model extreme shortfall. As we are interested in modeling the tails of the overall cost function, we now take the overall cost function *G* in (eq 1) as a random variable conditioned on ξ. For a prescribed confidence level α, the value-at-risk ν_α_(*G*) represents the lowest α-quantile of a random variable:

3 $$\nu_{\alpha }\left(G\right)=\inf\left\{G\in R:F_{G}\geq \alpha \right\}$$

where $F_{G}$ is the cumulative distribution function of *G*. A related quantity, the *Conditional* Value-at-Risk at level α, denoted as CVaR_α_(*G*), is then defined as the conditional expected value of *G* exceeding the specified quantile threshold:

4 $${\mathrm{CVaR}}_{\alpha }\left(G\right)=E\left[G|G\geq \nu_{\alpha }\left(G\right)\right]$$

where, among other risk metrics, CVaR has gained attention in the context of stochastic programming specifically,^46^ because it represents an asymmetric measure of variability, allowing one to focus on the tail of the cost distribution. Further, it is a coherent risk measure,^48^ thereby preserving convexity.

Given the above, the TSSP in (eq 1) can be extended to jointly optimize for the expected value and the CVaR using a mean-risk formulation^49^ with a weighting factor λ ≥ 0:

5 $$\underset{x\in X}{\min}E\left[G\left(x,\xi \right)\right]+\lambda {\mathrm{CVaR}}_{\alpha }\left[G\left(x,\xi \right)\right]$$

A standard and popular approach to solve TSSPs, either in the form of (eqs 1 or 5), is the Sample Average Approximation (SAA) method.^35^ SAA reformulates the distributional TSSP by sampling a finite set of possible realizations, or scenarios, ξ from the distribution P . Following this approach, the expected-value problem (eq 1) can be approximated by the corresponding SAA formulation (eq 6), representing a deterministic, but large-scale, monolithic problem defined over the sampled scenarios ξ_1_, ξ_2_, ..., ξ_*S*_, each associated with probabilities *p*_1_, *p*_2_, ..., *p*_*S*_:

6 $$\underset{x\in X,y_{s}\in Y\left(x,\xi \right)}{\min}c^{T}x+\overset{S}{\underset{s=1}{\sum }}p_{s}v\left(y_{s},x,\xi_{s}\right)$$

where copies of the second-stage variables *y*_*s*_ are now introduced according to the number of scenarios *S* to account for recourse action at each scenario ξ_*s*_. The polyhedral mapping that defines the feasible region Y(x,ξ) must also extend with the number of scenarios, i.e., $Y\left(x,\xi \right):X\times \Psi \to R^{n_{y}\times S}$ . Importantly, the feasible set must be enforced (i.e., the process model must be satisfied) for all second-stage scenarios *s* ∈ {1,···,*S*}, leading the SAA formulation to scale poorly with number of sampled uncertainty realizations.

For the risk-averse formulation (eq 5), an equivalent monolithic SAA formulation can be derived using some assumptions about convexity:^32,50^

$$\begin{array}{cc} \underset{x\in X,y_{s}\in Y\left(x,\xi \right),\nu ,\eta_{s}}{\min} & \left(1+\lambda \right)c^{T}x+\overset{S}{\underset{s=1}{\sum }}p_{s}v\left(y_{s},x,\xi_{s}\right)+\lambda \left(\nu +\frac{1}{1-\alpha }\overset{S}{\underset{s=1}{\sum }}p_{s}\eta_{s}\right)\\ \mathrm{s.t.} & \eta_{s}\geq 0,\forall s=\left\{1,...,S\right\}\\ \; & \eta_{s}\geq v\left(y_{s},x,\xi_{s}\right)-\nu ,\forall s=\left\{1,...,S\right\} \end{array}$$ 7

Here, η_*s*_ represents the excess second-stage cost of scenario *s* above the threshold ν (i.e., the value-at-risk at the confidence level α). Following Rockafellar and Uryasev,^32^ the auxiliary variables η_*s*_ are introduced for each scenario to enable the SAA risk-averse problem to be formulated as a monolithic optimization problem.

As the number of scenarios *S* in the SAA increases, the approximation of the underlying distribution improves. However, this comes at the cost of significantly larger optimization problems. Surrogate-based modeling approaches have gained increasing attention as one promising avenue to mitigate this scalability issue.

### 2.2 QNN Surrogate for the Second-Stage Objective

In general, there is a trade-off between computational efficiency and approximation accuracy, considering the continuous distribution of uncertainties in TSSPs. For example, considering SAA, as the discrete number of scenarios *S* increases, the approximation improves at the expense of additional model instances in the monolithic problem. This scaling becomes prohibitive in applications where many optimization runs are necessary or the deterministic optimization problem itself is already large.

As an alternative approximation, Alcántara et al.^43^ recently propose the use of Quantile Neural Networks (QNNs) as surrogate models for the second-stage value function. The QNNs are then embedded into the stochastic optimization problem as a set of mixed-integer linear constraints to generate a monolithic problem. The QNNs are trained to approximate distribution quantiles conditioned on the first-stage decisions (or a subset thereof). Using this surrogate approach foregoes the need to formulate copies of the model across discrete scenarios of the uncertain parameters; accordingly, the computational effort and approximation errors are shifted to the network training, which can be performed offline. Once trained, the QNN surrogate enables efficient solution of the stochastic optimization problem online. In particular, Alcántara et al.^43^ use incremental QNNs, or iQNNs, instead of the simpler QNNs, to avoid the “quantile crossing” phenomenon, where nonmonotonic behavior is observed across quantiles (i.e., the quantiles of the probability distribution should not overlap).

We now define $x={[\begin{array}{cc} X & Z \end{array}]}^{T}$ where $X\in R^{n_{X}}$ are the output-conditioning first-stage variables that act as inputs (features) for the iQNN as illustrated in Figure 2. For instance, the chosen plant capacity in *X* affects the recourse that can be taken, and therefore the second-stage value function. The remaining contextual variables $Z\in R^{n_{Z}}$ are assumed to be known and independent of *X*. The network outputs (labels) are the second-stage value function quantiles $Q_{\tau_{q}}\in R^{n_{Q}}$ where *n*_*Q*_ is a user-specified number of quantiles. This structure effectively learns a discretized distribution for the second-stage value function.

**Figure 2:**
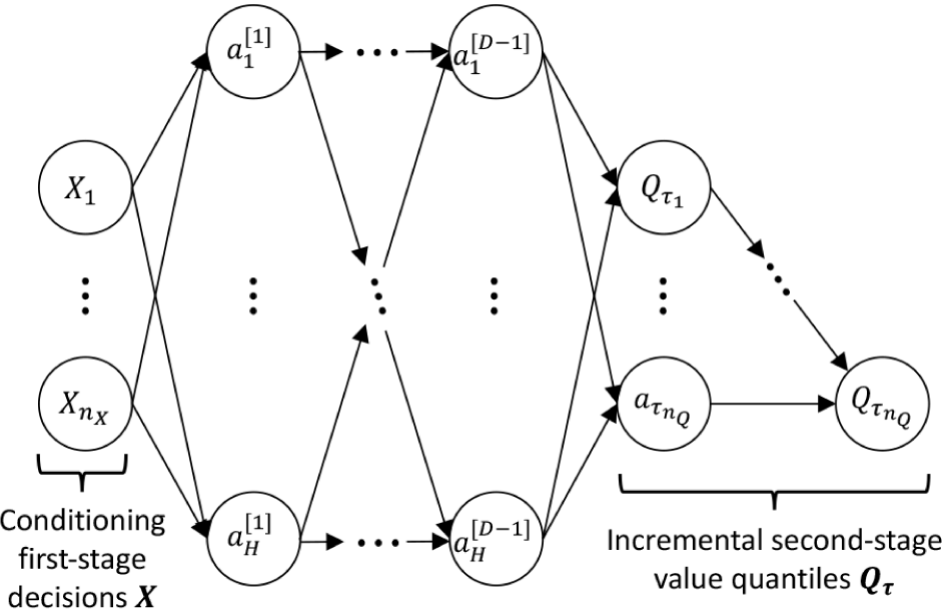
Architecture of second-stage surrogate iQNN.

A feedforward neural network of depth *D* and hidden-layer width *H* comprises the iQNN surrogate. We denote layer outputs $a^{[l]}\in R^{H_{l}}$ , biases $b^{[l]}\in R^{H_{l}}$ , and weights $W^{[l]}\in R^{H_{l}\times H_{l-1}}$ where *H*_*l*_ is the width of layer *l* ∈ {1,···,*D*}. The activation functions $g^{[l]}\left(\cdot \right):R^{H_{l}}\to R^{H_{l}}$ act on each layer as:

8 $$a^{[l]}=g^{[l]}\left(W^{[l]}a^{[l-1]}+b^{[l]}\right)$$

where the forward pass through the network is effectively a composition operator. In the context of TSSP, this formulation is specified such that *a* ^[0]^ = *X* as shown in Figure 2, which enforces neural network inputs to be the first-stage variables that affect the second stage. To enforce incremental behavior, the network is structured such that the output layer width corresponds to the number of quantiles $a^{[D]}\in R^{n_{Q}}$ , and each output quantile is the summation of the *D* ^*th*^ layer output and the previous quantile value, expressed as

9 $$Q_{\tau_{q}}=\overset{q}{\underset{i=1}{\sum }}a_{i}^{[D]},\forall q\in Q$$

where $Q=\left\{1,...,n_{Q}\right\}$ is the set of all quantiles, and $a_{i}^{[D]}\forall i\in Q$ are the individual elements of the output layer. By taking a non-negative activation function in the final layer $g^{[D]}\left(\cdot \right):R^{n_{Q}}\to R_{+}^{n_{Q}}$ , the outputs $Q_{\tau_{q}}$ are guaranteed to be monotonically increasing. The full QNN structure, including the incremental output, is shown in Figure 2. This structure produces the overall mapping $Q\left(\cdot \right):R^{X}\to R^{n_{Q}}$ .

Training the iQNN requires a data set $D=\{\left({\mathrm{X̂}}_{i},{\mathrm{v̂}}_{i}\right){\}}_{i=1}^{N}$ of *N* label-feature pairs, where *X̂*_*i*_ corresponds to sampled first-stage variable values, while *v̂*_*i*_ represents the corresponding second-stage value function evaluated at a specific scenario realization ξ̂_*i*_. The data set therefore contains samples to simultaneously learn the mapping over both variations in *X* and the distribution over scenarios ξ. Data generation and sampling procedures for these variables are covered in Subsection [Sec sec4.2]. To accommodate quantile learning, we use the “pinball” loss function, as proposed by Steinwart and Christmann:^51^

10 $$\theta =\arg\min\frac{1}{n_{Q}N}\underset{q\in Q}{\sum }\overset{N}{\underset{i=1}{\sum }}\tau_{q}\epsilon_{i,q}I_{\epsilon_{i,q}\geq 0}+\left(1-\tau_{q}\right)\epsilon_{i,q}I_{\epsilon_{i,q}<0}$$

where $\epsilon_{i,q}={\mathrm{v̂}}_{i}-Q_{\tau_{q}}\left(X_{i};\theta \right)$ is the estimation error of sample *i* from a data set of size *N* when predicting the *q* ^*th*^ quantile of the second-stage value function *v*, and $I_{\epsilon_{i,q}\geq 0}$ is an indicator function equal to 1 if the error is non-negative, and 0 otherwise. The second indicator $I_{\epsilon_{i,q}<0}$ is defined analogously. This loss is computed using the data point *v̂*_*i*_ and the iQNN prediction $Q_{\tau_{q}}\left(\cdot \right)$ , which is parametrized by the weights and biases $\theta ={[\begin{array}{cccccc} W^{[1]} & ... & W^{[D]} & b^{[1]} & ... & b^{[D]} \end{array}]}^{T}$ .

After training, the learned iQNN must be embedded within the two-stage stochastic optimization problem. Several software tools for handling this embedding step are available^52–54^ and are reviewed by López-Flores et al.^55^ and Misener and Biegler.^56^ Here we take all activation functions to be ReLU and use the simple big-M mixed-integer formulation following Alcántara et al.^43^ Consider a layer with a single ReLU activation (i.e., *H*_*l*_ = 1),

11 $$a^{[l]}=\max\left\{0,W^{[l]}a^{[l-1]}+b^{[l]}\right\}$$

where the maximum operator is the sole source of nonlinearity. This activation can be reformulated using a big-M approach that yields the following constraints:^57,58^

12 $$a^{[l]}\geq W^{[l]}a^{[l-1]}+b^{[l]}$$

13 $$a^{[l]}\leq W^{[l]}a^{[l-1]}+b^{[l]}-M^{-}\left(1-\sigma \right)$$

14 $$0\leq a^{[l]}\leq M^{+}\sigma$$

15 σ∈{0,1}

where (eqs 12 and 13) correspond to the linear ReLU subdomain and (eq 14) corresponds to the zero ReLU subdomain. The binary auxiliary variable (σ) is defined in (eq 15) to model the on–off state, and valid big-M constants *M* ^–^ and *M* ^+^ must be chosen such that

16 $$M^{-}\leq W^{[l]}a^{[l-1]}+b^{[l]}\leq M^{+}$$

Accordingly, interval arithmetic can be used to bound the above inequality with given bounds for a trained network’s layer inputs $a_{i}^{[l-1]}\in \left[{\underline{a}}_{i}^{[l-1]},{\overset{¯}{a}}_{i}^{[l-1]}\right]\forall i\in \left\{1,...,H\right\}$ to define the big-M constants as follows:

$$M^{-}=\overset{H}{\underset{i=1}{\sum }}\left({\underline{a}}_{i}^{[l-1]}\max\left\{0,W_{i}^{[l]}\right\}+{\overset{¯}{a}}_{i}^{[l-1]}\min\left\{0,W_{i}^{[l]}\right\}\right)+b^{[l]}$$ 17

$$M^{+}=\overset{H}{\underset{i=1}{\sum }}\left({\overset{¯}{a}}_{i}^{[l-1]}\max\left\{0,W_{i}^{[l]}\right\}+{\underline{a}}_{i}^{[l-1]}\min\left\{0,W_{i}^{[l]}\right\}\right)+b^{[l]}$$ 18

where (eqs 17) and ([Disp-formula eq18]) bound the smallest and largest possible cumulative outputs of a layer, respectively. Importantly, we can generalize the constraints generated in (eqs 12–15) to a network with arbitrary depth and width by applying the reformulation to all activations, as will be shown in the following sections. We note that more advanced formulations are available and could potentially improve computational performance.^59–61^

In summary, we train a QNN composed of neurons as given in (eq 8) using the quantile loss function in (eq 10) to model the distribution over the second-stage value function, given values of the first-stage decision. Incremental behavior is enforced by structuring the output using (eq 9). The trained network is then reformulated as a MILP and embedded into the TSSP using (eqs 12–15) and (eqs 17 and 18), giving a single monolithic problem.

### 2.3 Characterizing the Trust Region

In general, the accuracy of machine learning models decreases at points that lie far from the training data set.^62^ This can pose a challenge when embedding machine learning surrogates into optimization problems as solutions are often located at the variable bounds, and optimization solvers may seek to artificially “exploit” inaccurate surrogate models. To address this, we use the ϵ-CH approach of Maragno et al.,^63^ which defines a trust region around the training data set. Specifically, given the data set of sampled first-stage inputs $D_{X}=\{{\mathrm{X̂}}_{i}{\}}_{i=1}^{N}$ , the convex hull *CH*(*D*_*X*_) of these points is taken as a trust region where the surrogate model is assumed to be accurate. However, enforcing strict adherence to *CH*(*D*_*X*_) may be overly conservative when using the surrogate for optimization. Therefore, the strict convex hull *CH*(*D*_*X*_) is relaxed to ϵ-*CH*(*D*_*X*_). This method introduces a tolerance ϵ around the data points, which can be selected to balance between the surrogate model accuracy and the potential conservatism of the resulting optimal solution. The ϵ-*CH*(*D*_*X*_) domain is represented by the set of constraints in (eq 19), where $\mu \in R^{N}$ formulates the convex hull as a weighted sum over samples, and $s\in R^{n_{X}}$ represents the deviation from the convex hull (defined here using the $L^{1}$ norm), bounded by ϵ:

19 $$\begin{array}{l} \overset{N}{\underset{i=1}{\sum }}\mu_{i}{\mathrm{X̂}}_{i}=X+s\\ \overset{N}{\underset{i=1}{\sum }}\mu_{i}=1\\ \mu_{i}\geq 0,,\forall i\in \left\{1,..N\right\}\\ \left|\right|s|{|}_{1}\leq \epsilon \end{array}$$

The introduction of ϵ-*CH*(*D*_*X*_) in the optimization problem increases the number of the variables, as the number of weights μ_*i*_ grows with data set samples. However, in our computational experiments, this led to only a moderate increase in the solution time, while yielding improved solution quality overall. Alternatively, (eq 19) can be formulated using only the vertices of the convex hull, or over a subset of training samples (e.g., selected via a column generation algorithm.^63^

### 2.4 QNN-Based Risk-Averse Stochastic Optimization

We now combine the above components to give our full framework for risk-averse stochastic optimization of energy storage systems. The trained iQNN surrogate described in Subsection [Sec sec2.2] is embedded into the risk-averse two-stage stochastic optimization problem presented in Subsection [Sec sec2.1] with the bounds given in Subsection [Sec sec2.3]. The resulting monolithic iQNN-based risk-averse two-stage stochastic optimization problem is formulated as

20 $$\underset{x\in X}{\min}\left(1+\lambda \right)c^{T}x+\frac{1}{n_{k}}\overset{n_{Q}}{\underset{q=1}{\sum }}Q_{\tau_{q}}+\frac{\lambda }{n_{Q}-n_{c}}\overset{n_{Q}}{\underset{q=n_{Q}-n_{c}}{\sum }}Q_{\tau_{q}}$$

21

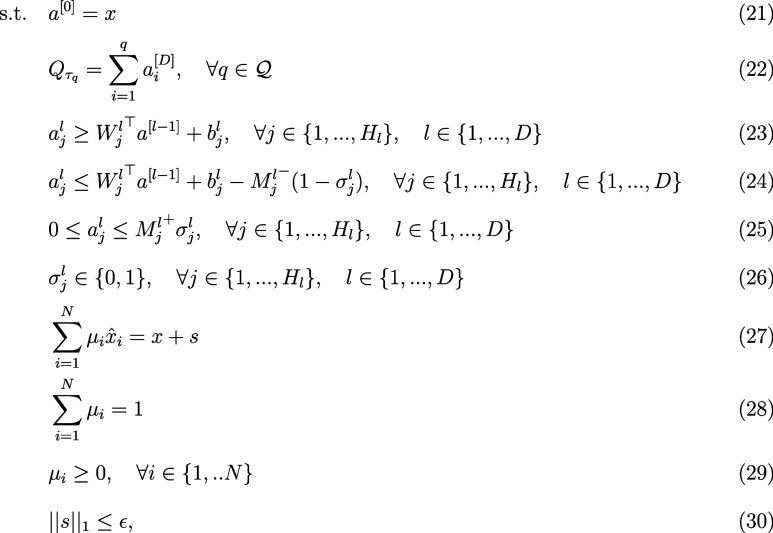

where $a_{j}^{l}\in R$ , $W_{j}^{l}\in R^{H_{l-1}}$ , and $b_{j}^{l}\in R$ denote, respectively, the output, weight vector, and bias of the *j* ^*th*^ neuron in the *l* ^*th*^ layer. Similarly for the big-M formulation, ${M_{j}^{l}}^{-}\in R$ , ${M_{j}^{l}}^{+}\in R$ , and $\sigma_{j}^{l}\in R$ denote, respectively, the big-M constants and the binary auxiliary variable. The objective (eq 20) corresponds to the two-stage objective function, where *n*_*c*_ is the number of right-tail quantiles used to approximate the CVaR. The constraint (eq 21) enforces the first-stage decisions to be taken as inputs to the iQNN, and (eq 22) enforces the incremental output of the QNN. The big-M reformulation of the ReLU activation functions is given in (eqs 23–26). Finally, (eqs 27–30) define the decision boundaries for the first-stage decision variables defined by the trust region for the iQNN surrogate.

This formulation is used to solve the two-stage stochastic optimization problem using an iQNN surrogate. The IHS process model, which defines the feasible regions for the problem X and Y , is presented next.

## 3 Integrated Hydrogen System Model

Hydrogen plays an important role in the (petro-)­chemical sectors, as it serves as a feedstock for several processes (e.g., Haber-Bosch, cracking) and for synthetic fuel production. It can also be used as an energy carrier and for energy storage owing to its high mass energy density and ease of electrochemical conversion. We consider production processes based on electrolyzers, which split water into oxygen and hydrogen using electricity. These hydrogen production systems can play an essential role in the energy transition, especially in sectors that are otherwise difficult to electrify.^4^

In this study, we focus on the optimal design and yearly operation of an integrated hydrogen production system (IHS) for the steel industry, based on the model described by Tsay and Qvist^25^ and depicted in Figure 3. In this setup, an electrolyzer produces hydrogen to satisfy the demand of a direct reduction iron (DRI) process, assumed to be continuous and constant. The hydrogen can either be heated and used directly in the DRI, or compressed and stored for later use. As an additional source of flexibility, a fuel cell can also be installed to convert the hydrogen back into electricity and sell it to the grid.

**Figure 3:**
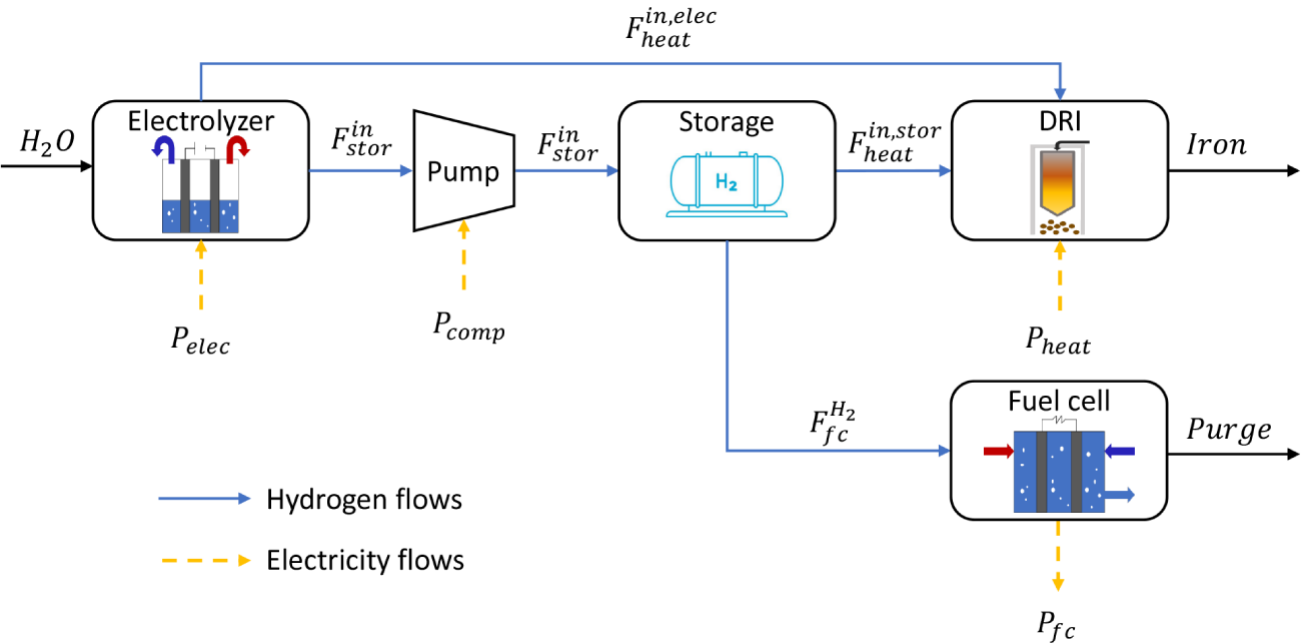
Schematic of the integrated hydrogen system. Adapted from Tsay and Qvist.^25^ Available under a CC-BY 4.0 license. Copyright [2023] Tsay and Qvist.

For systems that store energy, the evolution of the electricity price profile can deviate significantly from initial forecasts. To address this uncertainty, we employ a stochastic programming approach for the integrated design and operational optimization problem. The design problem comprises sizing the capacities of all process units, while the operational problem comprises creating the schedule for the year. The optimal solution(s) for both problems are affected by the true electricity prices. Accordingly, we formulate the integrated problem for the IHS as a two-stage stochastic optimization problem, using the framework from Section 2. The following sections describe the process model before formulating the resulting TSSP in Subsection [Sec sec3.6].

### 3.1 Electrolyzer

The model used here represents a generic electrolyzer parametrized by a fixed conversion efficiency rather than a more detailed model representing one specific electrolyzer technology; this was done to keep the results technology-agnostic and representative of the broader potential for electrolytic hydrogen production. The electrolyzer produces a hydrogen flow $F_{\mathrm{elec}}^{H_{2}}\left(t\right)$ [kg/h] by consuming DC power $P_{\mathrm{elec}}^{DC}\left(t\right)$ [MW]. The relationship between these is modeled proportionally by (eq 31) and depends on the base efficiency of the electrolyzer *L*_elec_ [-] and its degradation coefficient $L_{\mathrm{elec}}^{\deg}$ [-]:

31 $$F_{\mathrm{elec}}^{H_{2}}\left(t\right)=\frac{L_{\mathrm{elec}}^{\deg}}{L_{\mathrm{elec}}}\cdot P_{\mathrm{elec}}^{DC}\left(t\right),\forall t\in T$$

The connection to the electrical grid is represented using a linear scaling law to account for the AC/DC conversion in the rectifier. An efficiency factor $L_{\mathrm{elec}}^{AC/DC}$ [-] is used to model this conversion, while $L_{\mathrm{elec}}^{\mathrm{aux}}$ [-] considers the auxiliary equipment energy consumption. The total AC power imported from the grid $P_{\mathrm{elec}}\left(t\right)$ [MW] can be therefore computed using (eq 32):

32 $$P_{\mathrm{elec}}\left(t\right)=\left(L_{\mathrm{elec}}^{AC/DC}+L_{\mathrm{elec}}^{\mathrm{aux}}\right)\cdot P_{\mathrm{elec}}^{DC}\left(t\right),\forall t\in T$$

The connectivity of the electrolyzer with the other components of the plant is modeled using the mass balance in (eq 33). The total amount of produced hydrogen $F_{\mathrm{elec}}^{H_{2}}\left(t\right)$ can either be sent to the heating unit $F_{\mathrm{heat}}^{\mathrm{in},\mathrm{elec}}\left(t\right)$ [kg/h] for direct use in the DRI or to storage $F_{\mathrm{stor}}^{in}\left(t\right)$ [kg/h] for later use:

33 $$F_{\mathrm{elec}}^{H_{2}}\left(t\right)=F_{\mathrm{stor}}^{\mathrm{in}}\left(t\right)+F_{\mathrm{heat}}^{\mathrm{in},\mathrm{elec}}\left(t\right),\forall t\in T$$

To ensure consistency between design and operating decisions, the DC power consumed by the electrolyzer is constrained by the capacity *CAP*_elec_ [MW], which is modeled as a continuous variable in the optimization problem:

34 $$P_{\mathrm{elec}}^{DC}\left(t\right)\leq {CAP}_{\mathrm{elec}},\forall t\in T$$

We note that in some settings, discrete capacities may be required, e.g., to consider scaling up by number of electrolyzers. The upper bound on the electrolyzer capacity is defined in (eq 35), which expresses the maximum capacity in terms of the electricity $P_{\mathrm{elec},\mathrm{DRI}}^{\mathrm{DC}}$ [MW] needed to meet the baseload DRI hydrogen demand. The parameter δ_elec_ [-] determines the degree of flexibility of the plant; a larger value of δ_elec_ allows for more excess hydrogen production over the nominal DRI demand. When coupled with storage, this additional capacity enables the plant to leverage electricity price fluctuations.

35 $${CAP}_{\mathrm{elec}}\leq \delta_{\mathrm{elec}}\cdot P_{\mathrm{elec},\mathrm{DRI}}^{\mathrm{DC}}$$

### 3.2 Electric Heater

In DRI processes, the hydrogen feed must be heated to high temperatures. To account for this thermal energy requirement, we model an electric heater in the plant. Despite potentially lower efficiency, an electric heater is chosen to reflect a fully electricity-powered process, e.g., to enable complete integration with the power grid. The total hydrogen flow rate [kg/h] entering the heater includes flows from both the storage system $F_{\mathrm{heat}}^{\mathrm{in},\mathrm{stor}}\left(t\right)$ , and the electrolyzer $F_{\mathrm{heat}}^{\mathrm{in},\mathrm{elec}}\left(t\right)$ . These flows are related to the consumed electric power *P*_heat_(*t*) [MW] by (eq 36), depending on the heater efficiency η_heat_ [-], the specific heat capacity *c*_*p*_ [J/kg/K], and the temperature increase across the heater Δ*T*_heat_ [K]:

36 $$P_{\mathrm{heat}}\left(t\right)=\frac{\left(F_{\mathrm{heat}}^{\mathrm{in},\mathrm{elec}}\left(t\right)+F_{\mathrm{heat}}^{\mathrm{in},\mathrm{stor}}\left(t\right)\right)\cdot c_{p}\left(T_{heat}\right)\cdot \Delta T_{heat}}{\eta_{\mathrm{heat}}},\forall t\in T$$

Here, the heater efficiency is assumed to be 75%, while Δ*T*_heat_ is assumed constant for both streams coming from the storage and the electrolyzer, with an inlet temperature of 298 K and an outlet temperature of 1100 K. The specific heat capacity is evaluated using data from the NIST Chemistry WebBook^64^ at the average temperature *T*_*heat*_.

The total heater power demand is constrained by the installed capacity *CAP*_heat_ [MW], as given by (eq 37). Additionally, the total hydrogen flow to the heater must satisfy the demand *D*_DRI_ [kg/h] from the downstream DRI process, as enforced by (eq 38).

37 $$P_{\mathrm{heat}}\left(t\right)\leq {CAP}_{\mathrm{heat}},\forall t\in T$$

38 $$F_{\mathrm{heat}}^{\mathrm{in},\mathrm{elec}}\left(t\right)+F_{\mathrm{heat}}^{\mathrm{in},\mathrm{stor}}\left(t\right)\geq D_{\mathrm{DRI}},\forall t\in T$$

### 3.3 Storage System

There are several technologies available for hydrogen storage, including liquefaction, chemical conversion, and compression-based methods. In this work, we consider a straightforward configuration comprising a compressor and a storage unit (e.g., an underground storage cavity). The operation of the storage system is primarily characterized by two key variables: the hydrogen inventory level *I*_stor_(*t*) [kg] and the storage pressure *p*_stor_(*t*) [MPa]. The inventory level evolves over time and is modeled using a material balance between hydrogen inflows and outflows, $F_{\mathrm{stor}}^{\mathrm{in}}\left(t\right)$ and $F_{\mathrm{stor}}^{\mathrm{out}}\left(t\right)$ [kg/h]:

$$I_{\mathrm{stor}}\left(t\right)=I_{\mathrm{stor}}\left(t-1\right)+\left(F_{\mathrm{stor}}^{\mathrm{in}}\left(t\right)-F_{\mathrm{stor}}^{\mathrm{out}}\left(t\right)\right)\Delta t,\forall t\in T\backslash \left\{t_{0}\right\}$$ 39

To characterize the boundary storage operation, we consider a cyclic variation of the inventory level. This is achieved in the optimization by penalizing deviations between the inventory levels at the beginning and the end of each cycle, defined as the optimization period. This penalty helps ensure that optimization results do not derive artificial benefits by depleting initial storage levels.

In addition to the inventory level, the storage pressure is a key operational variable. It must remain within specified lower and upper bounds, $p_{\mathrm{stor}}^{LB}$ and $p_{\mathrm{stor}}^{UB}$ [MPa] to satisfy safety and operational requirements:

40 $$p_{\mathrm{stor}}^{LB}\leq p_{\mathrm{stor}}\left(t\right)\leq p_{\mathrm{stor}}^{UB},\forall t\in T$$

To model the pressure profile over time, we adopt a linearized version of the ideal gas law, given in (eq 41). The storage pressure depends on the compressibility factor *Z*_stor_ [-], the ideal gas constant *R* ^*H*2^ [J/mol/K], and the gas density ρ_stor_(*t*) [kg/m^3^]. The storage is assumed to operate isothermally at an ambient temperature of 298 K, while the compressibility factor *Z*_stor_ is evaluated at the midpoint between $p_{\mathrm{stor}}^{LB}$ and $p_{\mathrm{stor}}^{UB}$ . Tsay and Qvist^25^ found these approximations to be generally accurate for similar operational optimization settings, albeit for deterministic cases.

41 $$p_{\mathrm{stor}}\left(t\right)=p_{\mathrm{stor}}^{LB}+Z_{\mathrm{stor}}R^{H_{2}}\rho_{\mathrm{stor}}\left(t\right)T_{\mathrm{stor}},\forall t\in T$$

The gas density ρ_stor_(*t*) is calculated as the ratio of the inventory level *I*_stor_(*t*) to the storage volume *V*_stor_ [m^3^]­(eq 42). To formulate the overall model as a set of linear constraints, we can apply the McCormick envelopes^65^ to relax the feasible set of the nonlinear constraint (eq 42). When this relaxation is used, the original constraint (eq 42) is used to compute true values as a postprocessing step.

42 $$\rho_{\mathrm{stor}}\left(t\right)=\frac{I_{\mathrm{stor}}\left(t\right)}{V_{\mathrm{stor}}}\;\forall t\in T$$

The maximum volume of the storage unit is used to specify the installed capacity *CAP*_stor_ [kg], taking the hydrogen density at the maximum storage pressure effectively as the conversion factor between units.

43 $$V_{\mathrm{stor}}\leq \frac{{CAP}_{\mathrm{stor}}}{\rho \left(T_{\mathrm{stor}},p_{\mathrm{stor}}^{UB}\right)}$$

Finally, we model the allocation of hydrogen exiting the storage unit among the downstream components of the system. The total outflow $F_{\mathrm{stor}}^{\mathrm{out}}\left(t\right)$ [kg/h] is distributed between the fuel cell $F_{fc}^{H_{2}}\left(t\right)$ and the heater used in the DRI process $F_{\mathrm{heat}}^{\mathrm{in},\mathrm{stor}}\left(t\right)$ . This material balance is enforced through the following constraint:

44 $$F_{\mathrm{stor}}^{\mathrm{out}}\left(t\right)=F_{fc}^{H_{2}}\left(t\right)+F_{\mathrm{heat}}^{\mathrm{in},\mathrm{stor}}\left(t\right),\forall t\in T$$

### 3.4 Compressor

The storage of hydrogen requires compression from the electrolyzer outlet pressure *p*_elec_, which is assumed to operate at ambient conditions of 1 MPa to the storage pressure *p*_stor_(*t*). This compression process is modeled as a single-stage isothermal operation at *T*_comp_. The compression power *P*_comp_(*t*) [MW] depends on the hydrogen inflow rate $F_{\mathrm{stor}}^{\mathrm{in}}\left(t\right)$ in (eq 45), derived by Tsay and Qvist^25^ from a first-order Taylor expansion of the isothermal compression around a reference flow rate $F_{\mathrm{stor}}^{\mathrm{in}}=0$ kg/h and the midpoint of the storage pressure bounds $p_{\mathrm{stor}}\approx \frac{p_{\mathrm{stor}}^{LB}+p_{\mathrm{stor}}^{UB}}{2}$ . The authors also study the errors introduced by this approximation.

45 $$P_{\mathrm{comp}}\left(t\right)=\frac{R^{H_{2}}T_{\mathrm{comp}}}{\eta_{\mathrm{comp}}}F_{\mathrm{stor}}^{\mathrm{in}}\left(t\right)\ln\left(\frac{p_{\mathrm{stor}}^{LB}+p_{\mathrm{stor}}^{UB}}{2p_{\mathrm{elec}}}\right),\forall t\in T$$

As with other system components, the compressor operation must be consistent with the designed capacity *CAP*_comp_ [MW]:

46 $$P_{\mathrm{comp}}\left(t\right)\leq {CAP}_{\mathrm{comp}},\forall t\in T$$

### 3.5 Fuel Cell

Hydrogen fuel cells generate electricity and water by reacting hydrogen with oxygen, effectively reversing the electrolysis process. We model this similarly to the electrolyzer using a linear relationship (eq 47) between the DC power output of the fuel cell $P_{fc}^{DC}\left(t\right)$ [MW], and the hydrogen consumption rate $F_{fc}^{H_{2}}\left(t\right)$ [kg/h]. The relation depends on Faraday’s constant *F* [C/mol], the molar mass of hydrogen *M* ^*H*_2_^ [kg/mol], the cell voltage *V* [V], and the degradation factor $L_{fc}^{\deg}$ [-], which accounts for performance losses over time:

47 $$P_{fc}^{DC}\left(t\right)=2F\frac{F_{fc}^{H_{2}}\left(t\right)}{M^{H_{2}}}VL_{fc}^{\deg},\forall t\in T$$

The conversion from DC to AC power is modeled using (eq 48). This conversion accounts for the inverter efficiency $L_{\mathrm{fc}}^{DC/AC}$ [-] and auxiliary power losses $L_{\mathrm{fc}}^{aux}$ [-]:

48 $$P_{fc}\left(t\right)=\left(L_{\mathrm{fc}}^{DC/AC}-L_{\mathrm{fc}}^{aux}\right)P_{fc}^{DC}\left(t\right),\forall t\in T$$

Finally, the fuel cell output must respect the installed generation capacity *CAP*_*fc*_ [MW], as enforced by (eq 49):

49 $$P_{fc}\left(t\right)\leq {CAP}_{fc},\forall t\in T$$

### 3.6 Objective Function and TSSP Formulation

The objective function of the optimization problem accounts for the fixed and variable costs associated with the IHS, in order to minimize costs of delivering the hydrogen feed. The fixed cost *C*_fixed,*c*_ for each component c∈C = {elec, stor, heater, compressor, fc} depends on the respective installed capacity CAP_*c*_, the fixed operating cost *O*_*c*_, the per-capacity capital cost *C*_cap,*c*_, and the annualization factor *W*_*c*_. The annualization factor is calculated based on the component’s lifespan and the Weighted Average Cost of Capital. The fixed cost for each component is therefore computed as

50 $$C_{\mathrm{fixed},c}={\mathrm{CAP}}_{c}\cdot \left(C_{\mathrm{cap},i}\cdot W_{c}+O_{c}\right),\forall c\in C$$

The variable costs are evaluated based on the net power consumed or produced by each component *P*_c_(*t*) and the corresponding electricity price *c*_el_(*t*). For each time step *t*, the variable cost for process component *c* is given by

51 $$C_{\mathrm{var},c}\left(t\right)=P_{c}\left(t\right)\cdot c_{\mathrm{el}}\left(t\right),\forall c\in C,\forall t\in T$$

As mentioned previously, for the storage system, an additional penalty term is introduced to enforce cyclic operation:

$$C_{\mathrm{pen},\mathrm{stor}}\left(t\right)=\left|I_{\mathrm{stor}}\left(t_{0}\right)-I_{\mathrm{stor}}\left(t_{f}\right)\right|\cdot c_{\mathrm{pen}}$$ 52

This penalizes the difference between the initial *I*_stor_(*t*_0_) and final *I*_stor_(*t*_*f*_) inventory levels. While consistency with a hard-constraint approach can be ensured by properly tuning the penalty parameter *c*_pen_, the penalty-based approach used here provides more flexibility and can be used in systems where the strict satisfaction of the cyclic constraints in the second-stage might not be guaranteed for any first-stage decision. This property is important to guarantee the application of the proposed method, as for every possible realization of ξ, the set Y(x,ξ) should be nonempty (Subsection [Sec sec2.1]).

With all process models and cost components defined, we can now formulate the stochastic optimization problem, where the uncertainty is assumed to be introduced solely by the predicted electricity price profile ξ = *c*_el_(*t*) over the year. This formulation therefore helps mitigate risks associated with the “price-taker” formulation,^66^ i.e., treating electricity price as a fixed input. We approximate this as a TSSP and consider formulations both using the SAA (eq 6) and the iQNN surrogate (eqs 20–30), where the electricity price is assumed to be revealed for the rest of the year at a certain time *t*_obs_ (i.e., the system is built and the true electricity price trajectory is observed after some time in operation). Consequently, we consider a linear two-stage stochastic problem with uncertainty only in the objective cost coefficients. However, the QNN approach is model-agnostic, and the presented methodology can be adapted to other types of stochastic problems.^43^

This formulation represents an approximation of the real price-prediction setting, where forecasts become increasingly accurate closer to real-time, and therefore may be more accurately represented by a multistage formulation. However, we note that the TSSP satisfies the main motivation for the formulation: to consider that the IHS must be designed and built as the first stage, before the true electricity prices are observed in practice. Furthermore, the two-stage approach is more tractable, and our results in the case study show that the proposed formulation indeed produces significant improvement compared to the deterministic setting.

Given the two-stage setting, the first stage is represented by the investment planning and the operation of the system between the initial time *t*_0_ and the time *t*_obs_ when electricity prices are assumed to be observed. Therefore, the “here-and-now” variables include the capacity of the components, the storage inventory, and the power and flow operation of the components up to *t*_obs_. Meanwhile, the second-stage recourse decisions (“wait-and-see”) represent the operation from *t*_obs_ until the end of the time horizon *t*_*f*_ for the different scenario realizations. The distinction between first and second-stage variables for the IHS is summarized in Table 1. This decision structure was built into the model to enable more generality and accommodate for schemes such as flexible power purchasing agreements, where prices are negotiated at a set time. Furthermore, the horizon time *t*_*f*_ determines the operational time jointly optimized with the design. We consider the first year of operation to account for seasonal fluctuations, while practically limiting the size of the optimization problem. Considering a longer horizon would practically scale the number of scenarios required to accurately capture the uncertainty distribution, and increase the number of operational decision variables. Moreover, energy prices are difficult to predict in long horizons, and it is therefore common practice to consider uncertainties over one (representative) year.

**Table 1:** Summary of Main First-Stage and Second-Stage Decision Variables for the IHS Model

Stage	Variable	Description
First	*CAP*_c_	Installed capacity for c∈C
	*P*_c_(*t*)	Power profile for *c* = {elec, stor, heater, comp, fc} and *t* = {*t*_0_, ..., *t*_obs_}
	*I*_stor_(*t*)	Storage inventory for *t* = {*t*_0_, ..., *t*_obs_}
	*F*_c_(*t*)	Hydrogen flow for c∈C and *t* = {*t*_0_, ..., *t*_obs_}
Second	*P*_c_(*t*)	Power profile *c* = {elec, stor, heater, comp, fc} and *t* = {*t*_obs_, ..., *t*_*f*_}
	*I*_stor_(*t*)	Storage inventory for *t* = {*t*_obs_, ..., *t*_*f*_}
	*F*_c_(*t*)	Hydrogen flow for c∈C and *t* = {*t*_obs_, ..., *t*_*f*_}

## 4 Results and Discussion

We now apply of the methodology presented in Section 2 to solve the integrated design and the operational problem of the IHS, considering uncertainty in price forecasts, described in Section 3. Our analysis compares solutions using the iQNN-SP (eqs 20–30) and SAA formulations (eq 7) for solving the resulting TSSP. The comparison is performed in both risk-neutral and risk-averse settings.

In the following sections, we evaluate the performance of the iQNN approach relative to the SAA method in terms of solution quality and computational time across two different case studies. Specifically, we explore two cost parametrizations for the electrolyzer and fuel cell, reflecting potential changes in technology costs for key process components accompanying the energy transition. We consider this as a parametric study, motivated by the setting of a process operator seeking to design an operate an available technology. An alternative approach could instead consider these process parameter as (additional) sources of uncertainty.

The parameter values used in our case studies are first described in Subsection [Sec sec4.1], where we outline the technical parameters of the IHS, the main cost components of the plant, and the assumed uncertainty set. In Subsection [Sec sec4.2] we describe the data generation procedure and the iQNN training, while in Subsections [Sec sec4.3] and 4.4 we present the results of the two case studies. All computational experiments were performed on an Apple M4 Pro CPU using Pytorch 2.7.1 ^67^ for QNN training and Gurobi 12.0.2 ^68^ for modeling and solving the optimization problems.

### 4.1 Process Parameters

The components of the IHS are characterized by technical parameters including efficiencies, degradation factors, and operating conditions (e.g., temperatures and pressures). The parameters adopted in this case study are based on those reported by Tsay and Qvist,^25^ as summarized in Table 2.

**Table 2:** IHS Technical Parameters

	Parameter	Description	Value
Electrolyzer	$L_{\mathrm{elec}}^{AC/DC}$	Rectifier efficiency (inverse)	105%
	$L_{\mathrm{elec}}^{aux}$	Auxiliary power consumption	5%
	*L*_elec_	Base efficiency	0.05 MW/kg
	$L_{\mathrm{elec}}^{deg}$	Degradation factor	91.42%
	δ_elec_	Capacity bound factor	2.5
Heater	Δ*T*_heat_	Temperature drop	802 K
	η_heat_	Heater efficiency	75%
	*D*_DRI_	DRI demand	150 ton/h
Storage	*Z*_*stor*_	Compressibility factor	1.07
	$p_{\mathrm{stor}}^{lB}$	Pressure lower bound	2 MPa
	$p_{\mathrm{stor}}^{UB}$	Pressure upper bound	20 MPa
Compressor	η_comp_	Efficiency	70%
Fuel cell	$L_{\mathrm{fc}}^{DC/AC}$	Inverter efficiency	95%
	$L_{\mathrm{fc}}^{aux}$	Auxiliary power consumption	5%
	$L_{\mathrm{fc}}^{\deg}$	Degradation factor	91.42%
	*V*	Voltage	0.7 V

Our nominal capital costs are adopted from Tsay and Qvist;^25^ however, we perform sensitivity analysis on some key cost parameters as explained next. We consider a capital cost of 2€/kW for storage and 50€/kW for both the compressor and heater. Fixed operating costs range from 1 to 10€/kW/year across all components. The annualization factor is set at 0.08 for auxiliary equipment, 0.10 for storage, and 0.13 for all other components. We use the annualization factors calculated in Tsay and Qvist^25^ based on component lifespans and their weighted average cost of capital.

In Case Study 1, the electrolyzer and fuel cell are assigned a capital cost of 300€/kW and an auxiliary equipment cost of 500€/kW, with a fixed operating cost of 4€/kW/year. The cost of these technologies may decrease with increased innovation and scale-up in the current energy transition. To reflect potential cost reductions related to technological advancements, Case Study 2 explores a scenario where these costs are halved: 150€/kW for the main units, 250€/kW for auxiliary equipment, and 2€/kW/year for fixed operating expenses.

Over a yearly time horizon, the trajectory of the electricity price is uncertain, representing the random variable ξ in the stochastic problem. To generate realistic price scenarios, we leverage historical data and the fitted forecasting models from the PyPSA open-source unit commitment tool.^69^ The historical Day-Ahead Market prices are obtained from the platform of the German Transmission System Operator, covering the years 2019, 2022, and 2023 (Figure 4), which represent relatively different weather and price scenarios. Based on this data set, Welfonder et al.^69^ fit an ARIMAX prediction model using the residual load forecast and a weekend indicator time series as exogenous variables. To account for seasonality without incurring the computational cost of seasonal ARIMAX models, additional exogenous variables of the form sin­(2π*t*/*T*) and cos­(2π*t*/*T*) are introduced for different periods *T*.^69^ The uncertainty distribution is assumed to correspond to this fitted model, and therefore the model is then sampled to generate the finite scenario set required by the stochastic optimization framework. We note that the QNN framework requires only samples for training, and therefore could be deployed given only prediction scenarios of electricity price trajectories rather than a complete distribution/prediction model. In this case, predicted trajectories could directly be used as the sampled scenarios ξ_1_,···,ξ_*S*_, though the learned distribution of second-stage costs would be dependent on the sample coverage.

**Figure 4:**
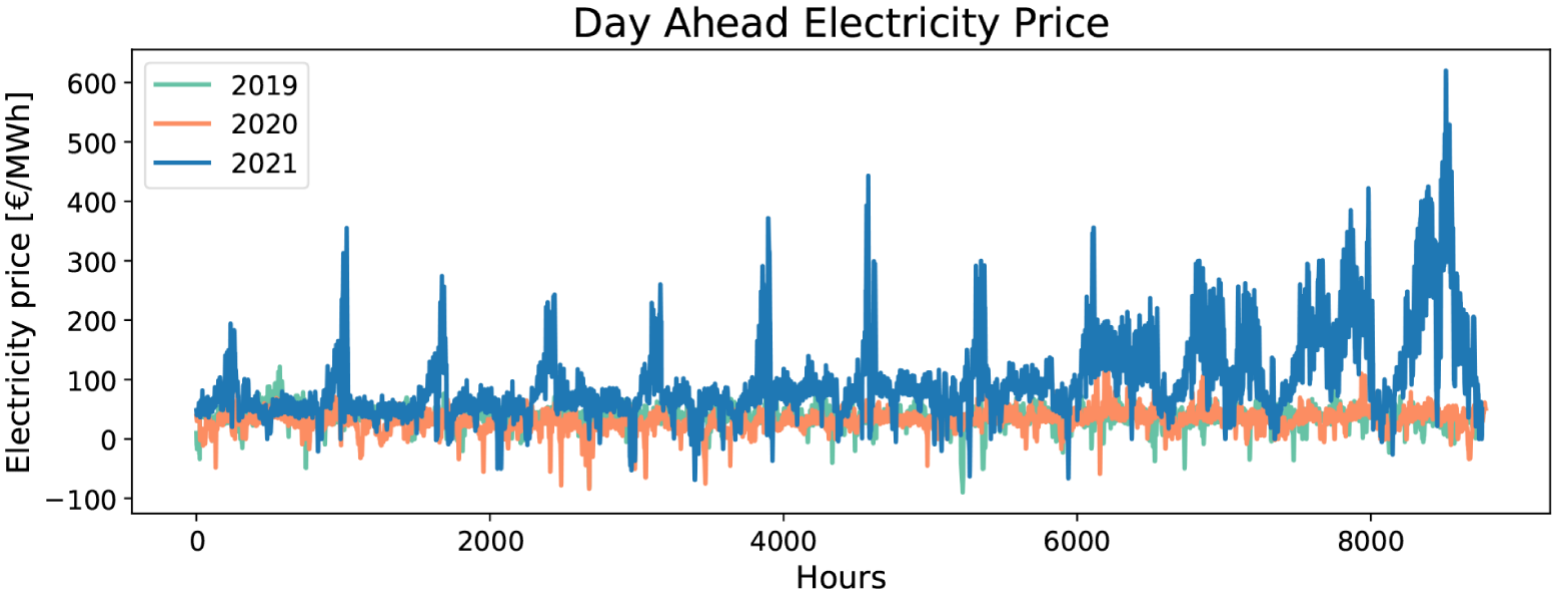
Historical electricity price series data from Welfonder et al.^69^

### 4.2 Data Generation and iQNN Training

For the integrated IHS problem, the first-stage inputs *X*_*i*_ that affect the second-stage problem are the installed capacity *CAP*_*c*_ of each component c∈C , along with the instantaneous storage inventory level *I*_stor_(*t*_*obs*_) at the time when uncertainty is assumed to be realized. Although other decision variables, such as the electricity consumption/production of the components before *t*_*obs*_, are included in the first stage of the stochastic problem, these variables are not used for the QNN training, as their state is independent of the second-stage value function to be learned by the surrogate model.

To generate training data, we randomly sample *X*_*i*_ from a uniform distribution defined over the support given by the variable bounds. For each sampled *X̂*_*i*_, the corresponding label *v̂*_*i*_ is obtained by solving the second-stage problem for a randomly selected scenario ξ̂_*i*_. This second-stage problem involves only a single scenario and is solved very quickly (usually in less than 1 s). This represents a key advantage of the QNN-based approach over others in the literature (such as NN-E in Neur2SP^41^), which require evaluating multiple scenarios per first-stage realization. As a result, the data set generation is easily parallelizable, substantially reducing the overall offline computational time. Specifically, we currently evaluate the scenarios in series, requiring several hours; however, the scenarios are entirely independent and can be solved in parallel. For example, in the limit case that 30,000 processing units are available, all scenarios could be simultaneously solved. For model training and hyperparameter tuning, we used a subset of 30,000 samples, with 24,000 for training (80%) and 6,000 (20%) for validation. The network architecture is limited to a single hidden layer and trained for 2,000 epochs with batch size of 256 using the Adam optimizer. A single hidden layer was chosen, as Alcántara et al.^43^ found that this architecture provided stable approximation accuracy, while deeper networks have a tendency to overfit. To tune the remaining hyperparameters, we perform^43^ a random search over 50 configurations where the learning rate and dropout rate are sampled from uniform distributions over [10^–5^, 10^–1^] and [0, 0.2], respectively. The number of neurons in the hidden layer is selected from the set {32, 64, 128, 256}, while the number of output neurons (corresponding to the number of predicted quantiles) is chosen from {50, 100}. The best configuration we obtained in terms of validation loss corresponds to 64 neurons in the hidden layer, 50 output quantiles, and 0.05 for dropout and 0.0005 learning rate. Training the model with this configuration requires 220.51 s on our machine, yielding a scaled training loss of 0.2602. The model was then evaluated on 10,000 test samples (25% of the full 40,000 samples data set), resulting in a test loss of 0.2618.

The trained surrogate iQNN was embedded using a value of ϵ = 0.5 (on a normalized basis) for the ϵ-*CH*(*D*_*X*_) in (eq 19). This value was empirically chosen to strike a balance between accuracy and quality of the solution, preventing extrapolation or overly conservative solutions. For a more systematic tuning approach, we refer to Alcántara et al.,^70^ where an iterative procedure is proposed to tune the trust region jointly with the ML training. In our experiments, all training data set samples were used to construct the convex hull for the trust region (eq 19). Even with all samples included, the solution time of the QNN remained very low compared to the SAA approximation. To improve the scalability, more efficient approaches such as the column generation method of Maragno et al.^63^ can be employed to select only the active vertices of the convex hull.

### 4.3 Case Study 1: Nominal Fixed Costs

Case Study 1 is characterized by (larger) nominal fixed costs of the electrolyzer and fuel cell. We consider the hourly operation of the plant over a year, while the observation time *t*_*obs*_ of the uncertain electricity price is set to the end of the first month. For instance, after observing electricity prices for a month, we are better able to guess which scenario is in place. We aim to assess the effectiveness of the iQNN-SP formulation for this problem compared to the SAA, which we consider as the baseline.

We first analyze this case study with traditional stochastic programming metrics. Specifically, we quantify the value of considering uncertainty in the IHS case study by comparing the solution of the recourse problem (RP) with the solution obtained by taking a purely deterministic approach. The RP is solved using the SAA formulation, using different numbers of scenarios. This preliminary study allows us to assess the impact of modeling uncertainty in the IHS case study and establish a suitable SAA baseline for comparing the surrogate-based method.

Next, we investigate the sensitivity of the recourse problem to the level of risk aversion and evaluate the quality of the iQNN-SP approximation and the computational time in both risk-neutral and risk-averse settings.

To support this, we determine a reasonable number of scenarios to include in the stochastic formulation. As noted previously, there exists a trade-off between SAA accuracy and computational time required to solve the resulting formulation. We therefore study how the objective function and computational time for the SAA vary with the number of scenarios in the risk-neutral setting. The lower and upper bounds for the recourse problem are given by the wait-and-see (WS) solution and the expected result of the expected value problem (EEV), respectively.^71^ Recall that the WS solution is given by $E_{\xi }\left[\underset{x\in X}{\min}G\left(x,\xi \right)\right]$ and represents the “oracle” case when uncertainties are perfectly known. The EEV evaluates the expected cost $E_{\xi }\left[G\left(\mathrm{x̅},\xi \right)\right]$ when the first-stage decisions are fixed to the optimal deterministic solution x̅ of the expected value (EV) problem, i.e., $\mathrm{x̅}=\arg\underset{x\in X}{\min}G\left(x,E\left[\xi \right]\right)$ . A summary of the models considered in the analysis is provided in Table 3.

**Table 3:** Overview of Models Considered in the Analysis

	Problem	Definition	Description
RP	Recourse Problem	(eq 1)	Two-stage stochastic program with recourse
SAA	Sample Average Approx.	(eq 6)	Finite-sample approximation of RP
iQNN-SP	iQNN Stochastic Program	(eqs 20–30)	QNN surrogate-based approximation of RP
WS	Wait-and-See	$E_{\xi }\left[\underset{x\in X}{\min}G\left(x,\xi \right)\right]$	Oracle benchmark with perfectly known uncertainty
EV	Expected Value	$\underset{x\in X}{\min}G\left(x,E\left[\xi \right]\right)$	Deterministic problem using expected parameters.
EEV	Expected result of EV	$E_{\xi }\left[G\left(\mathrm{x̅},\xi \right)\right]$	Expected cost when applying EV solution x̅ under uncertainty

Figure 5 (left) illustrates how the RP solution evolves when solved via SAA with different numbers of scenarios, while Figure 5 (right) reports the corresponding computational times. The scenarios generated following Welfonder et al.^69^ were sampled uniformly across the corresponding three original historical scenarios (Figure 4). We solve the SAA with up to 100 scenarios due to quickly increasing computational requirements, while we evaluate the WS and EEV bounds for the full set of 1000 scenarios to observe their asymptotic behavior. Figure 5 shows that the number of scenarios has a significant impact on computational time, while the objective value stabilizes after approximately 50 scenarios are included. Therefore, we adopt SAA with *S* = 50 scenarios as our baseline for further comparison, denoted as “SAA-50.”

**Figure 5:**
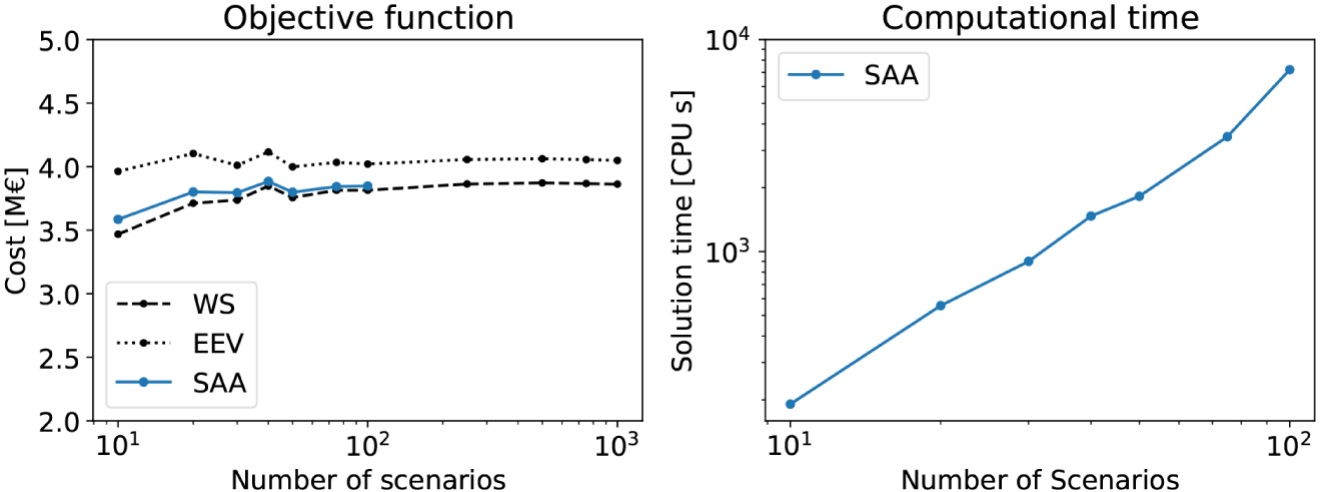
Case study 1 objective function (left) and computational time (right) of the recourse problem solved with the SAA formulation and different numbers of scenarios. The figure also shows the objective functions of the wait-and-see (WS) and expected result of the expected value problem (EEV) problems.

Another key insight is to analyze the gap between the EEV and the RP, known as the Value of the Stochastic Solution (VSS)^71^ and the gap between the RP and the WS, known as the Expected Value of Perfect Information (EVPI).^71^ The former quantifies the economic value of solving the stochastic optimization problem instead of optimizing based solely on expectations; the latter quantifies the economic gap with respect to an oracle with perfect foresight of the uncertainties. In short, the VSS and EVPI represent the economic benefit of stochastic optimization and perfect knowledge, respectively. Considering 50 scenarios, the VSS is approximately 0.2 M€, while EVPI is considerably smaller, namely 0.04 M€. This supports the use of stochastic modeling to explicitly incorporate uncertainty as a recourse problem. Although the TSSP approach represents an approximation of the (multistage) real world problem as noted previously, it significantly improves the decision-making process with respect to a deterministic approach (i.e., based on the EV problem).

With SAA-50 established as the baseline, we investigate the decision-making results under various levels of risk aversion, summarized in Table 4 and Figure 6. To model different levels of risk aversion, we consider a constant confidence level of 90% for the Conditional Value at Risk (eq 4) and vary the weight λ in the objective function (eq 5) between λ = 0, corresponding to a risk-neutral problem, and λ = 1, corresponding to a fully risk-averse setting. A sensitivity analysis to different confidence levels for the CVaR is included for both case studies as Supporting Information.

**Table 4:** Case Study 1 Results for Different Risk Weights *λ* and SP Solution Methods

	λ = 0		λ = 0.5		λ = 1	
	SAA	iQNN-SP	SAA	iQNN-SP	SAA	iQNN-SP
Objective function [M€]	3.799	3.871	6.871	6.965	9.931	10.055
True objective function [M€]	3.837	3.848	6.946	6.960	10.046	10.071
Expected cost [M€]	3.799	3.871	3.807	3.875	3.816	3.875
CVaR [M€]	-	-	6.127	6.18	6.115	6.18
Second stage cost [M€]	2.161	2.253	4.276	4.413	6.394	6.652
True second stage cost [M€]	2.198	2.229	4.355	4.408	6.51	6.667
Problem solution time [s]	1825.38	**3.46**	2927.56	**3.86**	1397.66	**4.03**
Model training time [s]	-	220.51	-	220.51	-	220.51
Electrolyzer capacity [MW]	16760	16883	17340	16858	17561	16858
Fuel cell capacity [MW]	0	95	0	96	0	96
Storage capacity [kg]	11035	9269	13886	13802	15320	13802
Compressor capacity [MW]	358	441	370	441	199	441
Storage level [kg]	5865	367	2319	796	2236	796

**Figure 6:**
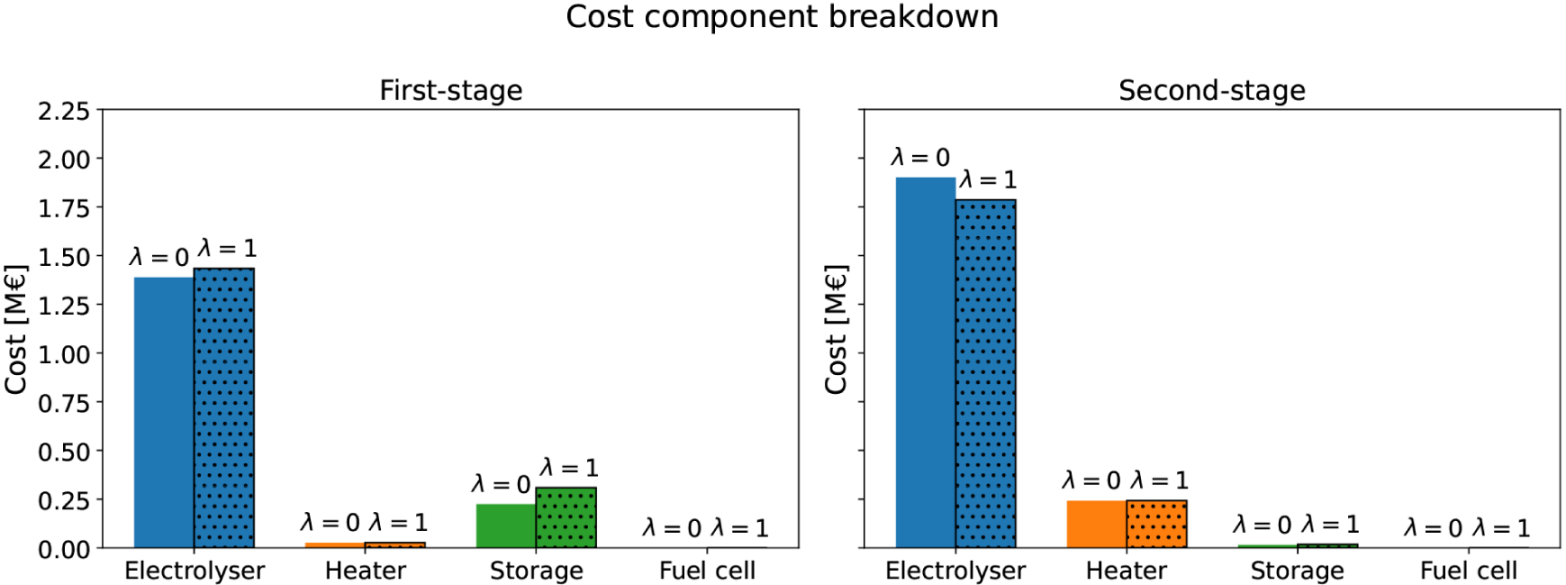
Case study 1 cost breakdown of the SAA solution for stages and components under different risk aversion levels (λ = 0 and λ = 1).

When λ = 0, the SAA solution installs an electrolyzer capacity of 16760 MW and a storage capacity of 11035 kg, with no fuel cell installed. The solution of the deterministic EV problem is instead 12535 MW for the electrolyzer and 3641 kg for the storage. Intuitively, we observe that accounting for uncertainty in the design and operational optimization results in increased capital investment to lower the overall cost by enabling more flexibility (as previously evidenced by the VSS gap). In other words, installing oversized equipment, and incurring more costs in the first stage, can offset larger future costs by providing added flexibility.

As the risk aversion weight λ increases, solutions involve installing even higher process capacities to hedge against high-operating-cost tail scenarios, with an electrolyzer capacity of 17561 MW and a storage capacity of 15320 kg for the case where λ = 1. Figure 6 shows the cost breakdown of the plant components for the first-stage and second-stage, given λ = 0 and λ = 1. Higher risk aversion leads to increased investment in the first-stage, resulting in a reduction of operating costs in the second stage. In other words, the more conservative decisions involve spending more on “here-and-now” fixed costs, in order to mitigate the effect of potentially risky scenarios that lead to extreme operational costs in the second stage. There is a cost associated with this risk mitigation: as reported in Table 4, the total expected cost increases with λ, while the corresponding CVaR decreases.

The iQNN-SP solutions exhibit similar trends to the SAA solutions. In the risk-neutral setting, the optimized installed capacities for the electrolyzer and storage are approximately 16883 MW and 9269 kg, respectively, while the optimal capacities for both increase as the risk-aversion weight λ increases to 1. However, the iQNN-SP approach results in solutions including the installation of a small fuel cell in all scenarios as shown in Table 4.

To compare the quality of decisions of the surrogate-approximated problems, we evaluate the true objective function over a ground-truth uncertainty set of 1000 scenarios, fixing the first-stage variables *x* to those obtained by SAA and iQNN. Table 4 compares the approximated and true objective values for different λ. We note that the iQNN-SP approximation is slightly more inaccurate than the SAA; however, this only amounts to a relative difference of less than 0.3%, meaning both approximations are relatively accurate to the ground-truth objective. Consequently, despite the differing configurations in storage level and fuel cell capacity suggested by SAA and iQNN-SP, their overall performance is similar, since the increased second-stage cost in iQNN-SP is balanced by a decrease in the first-stage.

Importantly, the problem solution time is significantly shorter, being <5 s for iQNN-SP, while always being >1300 s for the SAA-50. The iQNN approach even exhibits significantly faster solution times than SAA-10, as shown in Figure 5. This highlights that iQNN scales more efficiently, retaining computational advantages even when the number of SAA scenarios is very small (e.g., using fast-forward selection or clustering-based approaches). Therefore, the surrogate-method effectively shifts the computational burden offline. Furthermore, the iQNN model is independent of the risk-related parameters α and λ, requiring training only once to compute multiple solutions. Once the iQNN is trained, the surrogate-based method enables high-quality decisions to be obtained quickly, enabling applications such as parametric studies, online optimization, and/or counterfactual analyses under varying risk preferences.

### 4.4 Case Study 2: Reduced Fixed Costs

We now evaluate the impact of halved fixed prices for the electrolyzer and fuel cell, considering 150 €/kW for the main unit equipment and installation, 250 €/kW for auxiliary equipment, and 2 €/kW/year for fixed operating costs. This analysis explores the impact of potential reductions in technology costs as hydrogen electrolysis comes to industrial maturity. Additionally, it serves to assess the iQNN-SP approximation under a different incentive structure, i.e., a different ratio of fixed to operating costs. It also highlights the reusability of the trained iQNN: since data generation for training is independent of the first-stage parameters, the same iQNN model can be deployed.

The optimization results for Case Study 2 are given in Table 5 and Figure 7. Given the reduced fixed costs in this study, the model solutions tend to install large electrolyzer capacities even in the risk-neutral setting (Table 5 and Figure 7). For instance, the SAA solution, using 50 scenarios, installs 21785 MW of electrolyzer capacity and 13252 kg of storage. Intuitively, the lower capital costs allow for more equipment oversizing to mitigate second-stage costs. However, despite the lower costs, the fuel cell is still not installed. The incorporation of CVaR into the objective function primarily increases the storage size, which grows to 17180 kg at λ = 1, while the electrolyzer capacity remains nearly unchanged. This increased storage capacity enables sufficient hydrogen supply through periods of high electricity prices, decreasing the operating costs in the second stage as shown in Figure 7.

**Table 5:** Case Study 2 Results for Different Risk Weights *λ* and SP Solution Methods

	λ = 0		λ = 0.5		λ = 1	
	SAA	iQNN-SP	SAA	iQNN-SP	SAA	iQNN-SP
Objective function [M€]	3.097	3.23	5.802	6.002	8.499	8.771
True objective function [M€]	3.13	3.157	5.878	5.896	8.614	8.652
Expected cost [M€]	3.097	3.23	3.103	3.233	3.11	3.233
CVaR [M€]	-	-	5.398	5.539	5.389	5.539
Second stage cost [M€]	1.847	2.078	3.857	4.141	5.854	6.29
True second stage cost [Mg€]	1.881	2.004	3.933	4.032	5.971	6.168
Problem solution time [s]	886.61	**3.47**	2935.72	**3.84**	2217.1	**3.93**
Model training time [s]	-	220.51	-	220.51	-	220.51
Electrolyzer capacity [MW]	21785	21096	21785	21220	21785	21220
Fuel cell capacity [MW]	0	13	0	19	0	19
Storage capacity [kg]	13252	9227	15828	13807	17181	13807
Compressor capacity [MW]	290	443	290	443	290	443
Storage level [kg]	2137	527	2167	882	2062	882

**Figure 7:**
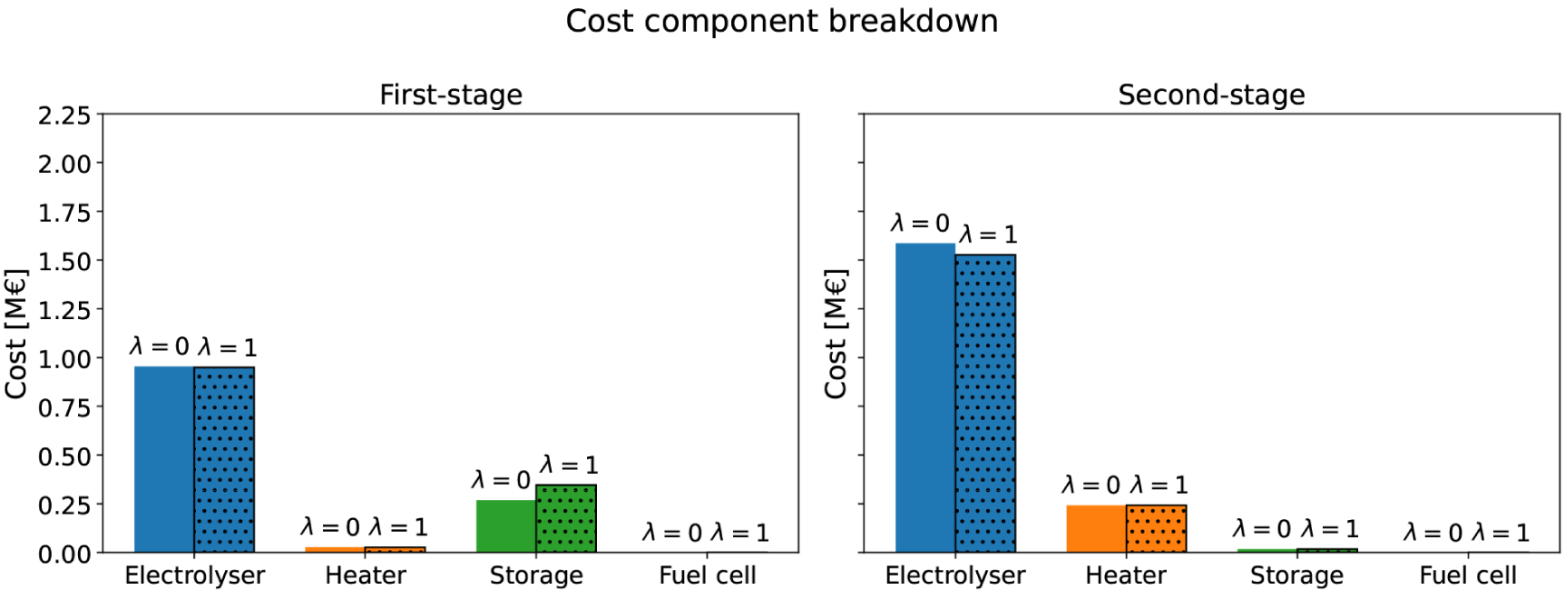
Case study 2 cost breakdown of the SAA for stages and components under different risk aversion levels (λ = 0 and λ = 1).

The iQNN-SP model captures similar trends, but solutions involve lower storage capacities overall. At λ = 0 an electrolyzer of 21096 MW and storage of 9227 kg are installed, while the optimal storage capacity is expanded to 13807 kg in the most risk-averse case λ = 1. Nevertheless, similarly to Case Study 1, the iQNN-SP installs the fuel cell with a small capacity in the range of 13–19 MW. By fixing the first-stage tactical decisions to the outputs of either the iQNN-SP or SAA models, we again compare their prescriptive performance by evaluating the true objective over the full 1000-scenario set (Table 5). The iQNN-SP exhibits slightly worse objective values than the SAA, but again both models have very similar accuracies, with differences remaining <1% across all risk-aversion levels. We again confirm that the iQNN-SP formulation offers significantly lower problem solution times. Therefore, this case study showcases the potential of embedding the same trained iQNN to efficiently solve multiple problems, enabling comprehensive what-if analyses under varying risk preferences and first-stage parameters assumptions.

## 5 Conclusion

In this work, we address the integrated optimal design and operation of energy-storage systems, using a large-scale integrated hydrogen system as a prototypical example. The plant produces hydrogen via an electrolyzer, with the option to install storage and a fuel cell to enhance system flexibility. The plant exchanges energy with the grid under time-varying electricity prices. To handle uncertainty in predictions at the design stage, we formulate the problem as a two-stage stochastic program, enabling both risk-neutral and risk-averse frameworks. We present a surrogate-based framework using Quantile Neural Networks (QNNs) to approximate the second-stage objective function. Unlike surrogate approaches that focus only on expectations, the QNN models the full distribution of second-stage costs, enabling the inclusion of risk metrics such as Conditional Value at Risk (CVaR).

We demonstrate our surrogate-based risk-averse stochastic optimization framework using two case studies, characterized by different fixed costs for the electrolyzer and the fuel cell. For both cases, including uncertainty, and additionally, CVaR in the objective formulation, increases the installed capacities of the electrolyzer and/or the storage system, increasing system flexibility and enabling hedging against high-electricity-cost scenarios. With decreased electrolyzer capital costs, a large electrolyzer capacity is installed even in the risk-neutral setting, and more risk-adverse formulations mainly suggest higher storage capacities. The case studies highlight the accuracy and computational efficiency of our proposed QNN-based framework, with problem-solving time two orders-of-magnitude shorter compared to the sample average approximation baseline, effectively moving the computational burden to offline training. Furthermore, we show how several analyses can be performed using the same trained QNN, making our framework a potentially powerful tool for what-if analyses under many counterfactuals constructed by varying risk tolerances and parameter sensitvity studies.

We identify a few directions for future work. From an application perspective, future analyses could include renewable energy production within the plant, enabling the production of “green” hydrogen and introducing additional sources of uncertainty in the energy supply. From a methodological perspective, the accuracy of the QNN for the prescriptive task can be enhanced, using adaptive data generation techniques in the training^42^ or decision-focused learning. Further, more complex QNN architectures could be explored to enhance the flexibility of the surrogate problem. Alternatively, this approach may be extended to a multistage setting by embedding multiple QNNs, given the flexibility of the method.

## Supplementary Material



## Notes

The results of a sensitivity analysis to CVaR confidence levels for both Case Studies 1 and 2 (PDF)
