# Supplementary material for: Integrated Design and Scheduling of Hydrogen Processes under Uncertainty: A Quantile Neural Network Approach
Source: Ind Eng Chem Res. 2025 Oct 22;64(44):21235–50. doi: 10.1021/acs.iecr.5c03288 (PMC12593336; doi:10.1021/acs.iecr.5c03288)
Supplement: Supplementary file 1 [file ie5c03288_si_001.pdf]

Supporting Information for "Integrated design and scheduling of hydrogen processes under uncertainty: a quantile neural network approach"

Lavinia M.P. Ghilardi,[†,‡] Gabriel D. Patrón,[†,‡] Antonio Alcántara,[¶] and Calvin Tsay[*,†,‡]

†*Department of Computing, Imperial College London, London, SW7 2AZ, UK*

‡*Centre for Process Systems Engineering, Imperial College London, London, SW7 2AZ, UK*

¶*Department of Statistics, University Carlos III of Madrid, Leganés, 28911, Spain*

E-mail: c.tsay@imperial.ac.uk

Table 1: Sensitivity study to CVaR parameters for Case Study 1

	$\lambda = 0$		$\lambda = 0.5$				$\lambda = 1$			
	SAA	iQNN-SP	$\alpha = 70$		$\alpha = 90$		$\alpha = 70$		$\alpha = 90$	
Objective function [M€]	3.800	3.871	6.778	6.879	6.871	6.965	9.748	9.882	9.931	10.055
Expected cost [M€]	3.800	3.871	3.806	3.875	3.807	3.875	3.813	3.875	3.816	3.875
CVaR [M€]	-	-	5.945	6.008	6.127	6.180	5.935	6.008	6.115	6.180
Solution time [s]	2413.12	3.42	1296.66	3.83	1263.97	3.86	873.75	3.41	1397.66	4.03
Electrolyzer capacity [MW]	16797	16883	17148	**16858**	17340	**16858**	17262	**16858**	17561	**16858**
Fuel cell capacity [MW]	0	95	0	96	0	96	0	96	0	96
Storage capacity [kg]	11042	9269	13650	**13802**	13886	**13802**	14864	**13802**	15320	**13802**
Compressor capacity [MW]	359	441	366	441	370	441	194	441	199	441
Storage level [kg]	2459	367	2463	796	2319	796	2400	796	2236	796

Table 2: Sensitivity study to CVaR parameters for Case Study 2

	$\lambda = 0$		$\lambda = 0.5$				$\lambda = 1$			
	SAA	iQNN-SP	$\alpha = 70$		$\alpha = 90$		$\alpha = 70$		$\alpha = 90$	
Objective function [M€]	3.097	3.230	5.717	5.918	5.802	6.002	8.330	8.603	8.499	8.771
Expected cost [M€]	3.097	3.230	3.102	3.233	3.103	3.233	3.107	3.233	3.110	3.233
CVaR [M€]	-	-	5.230	5.370	5.398	5.539	5.223	5.370	5.389	5.539
Solution time [s]	969.70	4.27	2721.83	4.16	2935.72	3.84	2275.83	3.55	2217.10	3.93
Electrolyzer capacity [MW]	21785	21096	21785	**21220**	21785	**21220**	21785	**21220**	21785	**21220**
Fuel cell capacity [MW]	0	13	0	19	0	19	0	19	0	19
Storage capacity [kg]	13252	9227	15558	**13807**	15828	**13807**	16665	**13807**	17181	**13807**
Compressor capacity [MW]	290	443	290	443	290	443	290	443	290	443
Storage level [kg]	2137	527	2212	882	2167	882	2212	882	2062	882
